# Exploring the Climate Temperature Effects on Settlement Intentions of Older Migrants: Evidence from China

**DOI:** 10.3390/ijerph19084896

**Published:** 2022-04-18

**Authors:** Hongjie Wang, Xiaolu Gao, Zening Xu, Yuan Li, Xinyue Zhang, Mark W. Rosenberg

**Affiliations:** 1 Key Laboratory of Regional Sustainable Development Modeling, Institute of Geographic Sciences and Natural Resource Research, Chinese Academy of Sciences, Beijing 100101, China; wanghj.16s@igsnrr.ac.cn (H.W.); zhangxy.18b@igsnrr.ac.cn (X.Z.); 2 College of Resources and Environment, University of Chinese Academy of Sciences, Beijing 100049, China; 3 Department of Geography and Planning, Queen’s University, Kingston, ON K7L 3N6, Canada; 17yl24@queensu.ca (Y.L.); mark.rosenberg@queensu.ca (M.W.R.); 4 China Institute for Urban Governance, Shanghai Jiao Tong University, Shanghai 200030, China; xuzening@sjtu.edu.cn

**Keywords:** temperature, older migrants, settlement intention, permanent migration

## Abstract

Permanent migration across provinces in China has become an important strategy for Chinese older people to respond to a temperature-unfriendly place of residence in late life. However, the relation between temperature effects and permanent settlements of older migrants remains unclear. Based on the data obtained from China Migrants Dynamic Survey, this paper examined how four temperature effects (i.e., cold effect, heat effect, temperature gap effect, and temperature zone effect) play a role in shaping older migrants’ intentions to settle permanently in a destination place by conducting logistic regression analysis. Our findings show that: (1) extreme cold (rather than extreme heat or mild temperature) was found to have significant effects on settlement intentions of older people; (2) relative winter temperature between origin and destination places rather than absolute winter temperature in the destination place has a significant positive effect on the settlement intentions; (3) spatially, older migrants tend to migrate to geographically adjacent temperature zones. Our findings will inform a more effective planning and allocation of services for supporting older people by better understanding trends and intentions of older migrants.

## 1. Introduction

The migration of older people has become a global demographic trend within developed and developing countries. In China, for instance, the population of older migrants increased from 9.34 million in 2010 to 17.78 million in 2015, and by 2015, the population accounted for 7.20% of the total migration populations and 8.01% of the total older populations. In particular, the spatial pattern of older migrants in China tends to shift from “rural-to-urban” to “urban-to-urban” and from “migrating within a province” to “migrating across provinces”. Whether to have local “Hukou” (Hukou is a legal document prepared by the Chinese Public Security Department to record and retain basic population information) is an important factor of permanent migration. Based on the Hukou status, some categorize older migrants into Hukou-transferred migrants and non-Hukou-transferred migrants. Some categorize older migrants into temporary migration, seasonal/regular migration [1], and permanent migration [2] according to the residence time in a destination. Typically, the planning and allocation of public services are designed on a basis of estimated populations at the local level. Therefore, older migrants are not only linked to improve individual wellbeing [3], but also to shape the spatial distribution of older populations as well as supply-and-demand of relevant resources (e.g., housing, amenities), especially for those who want to settle [4,5]. 

Existing studies on the settlement intention of older migrants focus mainly on developed countries. Migrations of older populations were found to be motivated by various factors, including high income, low living costs [6], healthcare access [7,8,9], age-friendly amenities [10], environment [11], and so on. In addition, Rallu believed that the willingness to settle may decrease as distance increases [12]. Handlos proposed that changes in health status motivated older migrants to settle or return [13]. For instance, Atkins identified two age peaks (55–64 and over 85) are likely to settle permanently in the destination area [14]. Place attachment is also an important factor in migration [15,16], which can improve satisfaction with the destination. Except for above factors, the willingness of Chinese older migrants to settle permanently is partly affected by a family arrangement [17,18] and Hukou [19], which is different from Western countries. For example, the purchase of real estate is not only associated with older people’s own preference of settlements but also with the opportunity of living close to where their adult children live and taking care of their grandchildren [20,21]. In addition, Hukou links with welfare services and has a damping effect on the settlement intention of older migrants if they face barriers to the services in a destination area without local Hukou [22,23].

Increasingly, climate is considered an important driver of migration, with the impact on living environments and, thus, on older people’s health. Climate may provide hundreds of millions of people with an additional incentive to migrate based on a global dataset [24,25]. Kaczan and Orgill-Meyer proposed that slow-onset climate changes are more likely to promote migrations than rapid-onset changes [26]. They believe that climate impacts migration in a nonlinear fashion, with the extent of household capability and vulnerability. Among the various climatic factors, temperature has been proved to be the principal factor. Mueller showed that heat stress, not rainfall, flooding, or moisture, is most strongly associated with migration, and the temperature by quartile has a nonlinear impact on migration in Pakistan [27]. Bohra-Mishra estimated a nonlinear relationship between temperature and migration in Indonesia, and the temperature effect is more pronounced than the rainfall effect [28]. Gray also strongly supported temperature effects on permanent migration rather than precipitation effects [29]. Adult migrants prefer destinations with small seasonal changes in temperature [30].

Temperature effects have significant impact on vulnerable groups of population especially those older migrants [31]. Some scholars proposed that heat waves can cause insomnia and fatigue [32,33,34], some suggested that cold spells will increase the risk of cardiovascular, cerebrovascular diseases, and fracture [35,36], and some demonstrated that there is a strong “U” relationship between temperature and mortality suggesting extreme cold or hot leads to higher death [37]. Consequently, migration to a temperature-friendly place has become an important strategy for older people. For example, Florida and California in the United States [38] and Hainan Island in China [39,40] are the main destinations for temporary or circular migration. However, research is limited on how temperature relates to the permanent migration of older migrants, that is, the willingness to settle. With the growth of “silver economy” [41,42,43], understanding the relationship between temperature and settlement intention of older migrants is critical to inform a design and planning of policies for meeting needs for productions and services for older people, especially for both “move-in” and “move-out” areas [44,45,46].

In general, this study aims to explore the temperature impact on settlement intentions of older migrants in China. First, understanding the settlement intentions of older migrants plays an important role in the allocation and provision of health care and other public services resources in “original” and “destination” areas when migration occurs. Second, extreme weather is becoming more frequent than before along the global climate change, with greater impact on older populations that are more vulnerable than younger populations. In particular, various temperature elements are characterized by geographical heterogeneity. Thus, there is need for understanding how the temperature elements vary in space and how these variations play a role in shaping the settlement intentions of older people. Finally, our findings can inform a better design of services policies by better understanding demographic changes because of permanent migrations of older populations. The findings also provide a nuanced understanding about temperature, which supports the coupled temperature-migration system studies.

## 2. Materials and Methods

### 2.1. Data

We obtained the data relevant to older migrants from the China Migrant Dynamic Survey (CMDS) conducted by the National Health Commission from 2009 to 2018. The latest CMDS data in 2018 does not include individual origin information, so this study used the CMDS data in 2017, which covers the spatial information of origin and destination. The survey sample used the annual report data of the migrant population of 31 provincial units in the previous year as the basic sampling frame and adopted a stratified, multi-stage, scale-proportional PPS method for sampling. The individual questionnaire mainly includes: (1) family members, income and expenditure; (2) employment; (3) mobility and settlement intention; (4) health and public services. Three principles were used in selecting samples for our analysis: respondents lived in the destination for 1 year or more, non-native floating population in prefecture-level cities (without local Hukou), and over 60 years old (born before 1957). The sample number of older migrants who met these features is 6478 in the 2017 CMDS database. 

### 2.2. Variables

#### 2.2.1. Dependent Variable

The dependent variable for this study is the settlement intentions of older migrants. Considering China’s unique “Hukou” system, there are two kinds of long-term settlement intentions for the older migrants: one is “Hukou transfer intention” meaning the Hukou status has changed, and the other is a broader “settle-down intention”. This paper selected broader “settlement intention” as the dependent variable, which was assessed based on the answer to the survey question, “How long do you expect to stay in the destination city if you plan to settle here?” in the CMDS. A binary variable of settlement intention was coded “1” for those answering, “willing to stay more than 5 years” and “0” for the other answers. In other words, the meaning of settlement intention in this paper was willing to stay in the destination city for more than 5 years. We identified those older people who intended to stay at a destination place in the next five years as older migrants for permanent settlement, taking into account life expectancy and the use of destination facilities.

#### 2.2.2. Key Independent Variable

**Figure 1:**
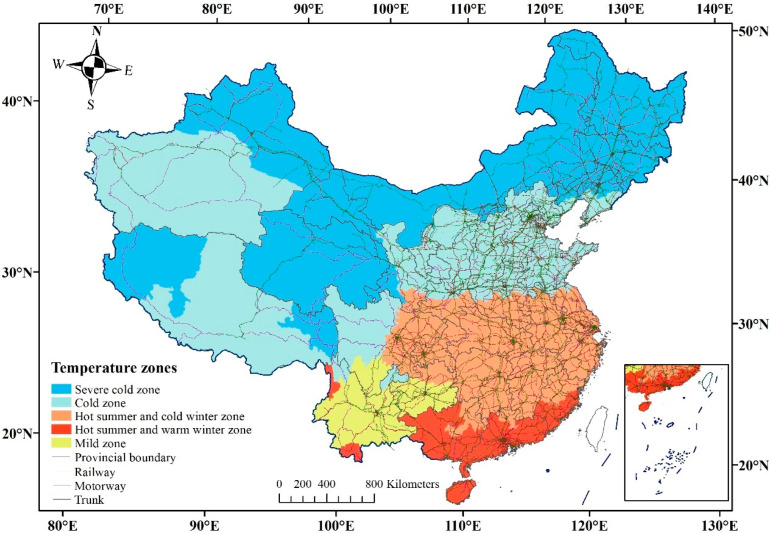
Temperature zones—building climate demarcation in China. Note: Transportation data were obtained from Open Street Map (https://www.openstreetmap.org/, accessed on 1 January 2021).

Four measurements of temperature effects were selected: cold effect, heat effect, temperature gap effect and temperature zone effect. The temperature observation data were obtained from China meteorological stations (https://www.resdc.cn/, accessed on 1 January 2021) and calculated by kriging interpolation, with the spatial resolution of about 1 km.

(1). Cold effect was expressed by Origin coldest-month average temperature (OCT) and Destination coldest-month average temperature (DCT), with three bins: −10 °C and below, −10 to 0 °C, 0 °C and above. Among them, the coldest month was January, the hottest month was July, and the specific temperature threshold was obtained according to the division index of temperature zone in 2017.
(2). Heat effect was represented by origin hottest-month average temperature (OHT) and destination hottest-month average temperature (DHT), with three bins: 23 °C and below, 23 to 28 °C, 28 °C and above.
(3). Temperature gap effect was showed by coldest-month temperature gap between origin–destination (GCT) and hottest-month temperature gap between origin–destination (GHT), in which GCT and GHT were divided into two categories based on 5 °C and 1 °C, respectively.
(4). Temperature zone effect was expressed by the spanning of temperature zones. Building climate demarcation divides China into five main temperature zones [47] as shown in Figure 1: severe cold zone, cold zone, hot summer and cold winter zone, hot summer and warm winter zone and mild zone. The “spanning” was used to link the origin temperature zone and the destination temperature zone, which were divided into three spatial relationships about similar zone, adjacent zones, and non-adjacent zones.

#### 2.2.3. Control Variables

We grouped control factors into four categories: individual factors, family factors, migration factors, and destination factors. The data of individual, family, and migration factors were obtained from CMDS in 2017 (as shown in Table 1), and the data for destination cities were acquired from the *China Statistical Yearbook* and various urban Statistical Yearbooks in 2016 due to the lag in migration.

(1) Individual factors included gender, age, education level, marital status, health status, and Hukou. Migration was described as a spontaneous personal or family behavior [48], so relatively capable and educated older migrants may tend to move [49]. Among them, marital status was divided into “married” for “first marriage, remarriage or cohabitation” and “single” for “unmarried, divorced or widowed”. Health status was divided into “healthy” for “healthy, basic healthy” and “unhealthy” for “unhealthy including able and not able to take care of themselves”.
(2) Family factors included the expenditure-income ratio and housing property. The expenditure–income ratio was the ratio of household expenditure to household income, which measured their economic and consumptive situation in the destination city. The housing property variable was divided into the self-owned house and non-self-owned house, in which self-owned house included self-purchased commercial housing, affordable housing, housing with limited property rights, and self-built housing.
(3) Migration factors included migration purposes, migration time, and migration distance. Migration purposes were classified as “economic purpose” for “working or business” and “non-economic purpose” for “enjoy life, being close to family, marriage, or other reasons”. Migration distance was the logarithmically normalized distance between origin and destination places.
(4) Destination city factors included city size, GDP per capita, and hospital beds per thousand people. Scholars had different views on the impact of city size on migrations. Some saw larger cities as more attractive [50] and others saw smaller cities as more attractive [51]. Destination city sizes were divided into four categories according to population sizes. GDP per capita and hospital beds per thousand people, respectively, reflected the economy and public services of the destination city.

### 2.3. Logistic Regression

The variety of migration models mainly included regression models [2], dynamic simulation models [52], life-course approach [53], decision tree models [54], and eigenfunction-based spatial filtering approach [22], and so on. In this paper, we adopted the logistic regression model. 

As the dependent variable, older migrants’ settlement intentions were classified as either “willing” or “unwilling” in CMDS. A binary logistic regression model served to fit these data and explored the impacts of temperature on settlement intentions. The model evaluated how independent variables affect the response probability of the dependent variable, represented as P when the dependent variable took values j ∈ [0,1]. The response probabilities could be written as:

(1) $$\mathrm{logit}\left(P\right)=\ln\frac{P}{1-P}\beta_{0}+\beta_{1}x_{1}+\beta_{2}x_{2}+\cdots +\beta_{n}x_{n}$$

(2) $$P=\frac{e^{\beta_{0}+\beta_{1}x_{1}+\beta_{2}x_{2}+\cdots +\beta_{n}x_{n}}}{1+e^{\beta_{0}+\beta_{1}x_{1}+\beta_{2}x_{2}+\cdots +\beta_{n}x_{n}}}$$

which denoted *P* as the probability of older migrants’ settlement intentions, $\beta_{0}$ as the intercept, $\beta_{i}$ as the estimated coefficients of the independent variables, and $x_{i}$ as the set of independent variables, and $\frac{P}{1-P}$ as the odds ratio of those willing to settle to those unwilling to settle. The binary logistic regression model was carried out by maximum likelihood estimation (MLE). If the odds ratio was greater than 1, then it indicated there was a relatively strong willingness to settle given the reference conditions.

## 3. Results 

### 3.1. Spatial Distribution

**Figure 2:**
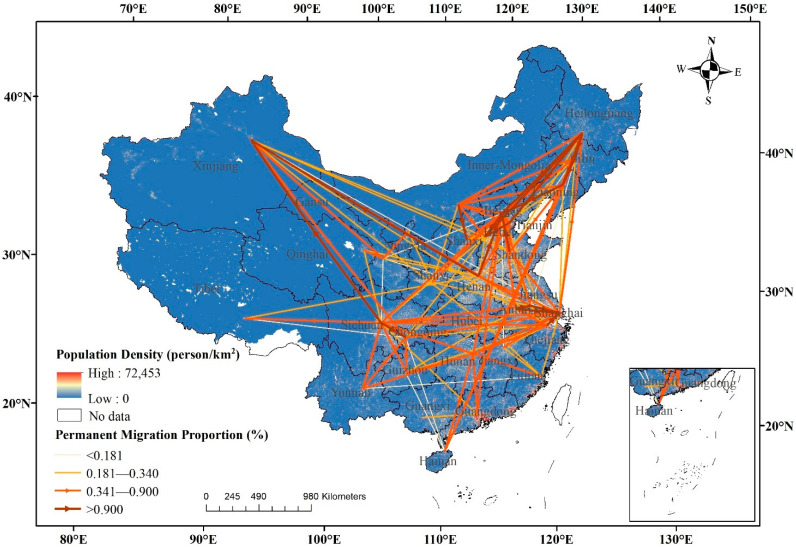
Migration flows of older migrants on the provincial scale in China in 2017. Note: The population density data were obtained from Resource and Environment Science and Data Center (https://www.resdc.cn/, accessed on 1 January 2021).

To visualize the older migration, we drew flow diagrams of migration and permanent migration on the provincial scale using ArcGIS. In the flow diagrams of migration proportion, we summed up the number of older migrants from province i to province j and calculated the proportion of older migrants to total samples for each flow. A thicker flow represents a higher migration proportion, a thinner flow represents a lower migration proportion, and the arrow represents the migration direction. It can be seen that there are many thicker flows in the migration pattern of older migrants from Figure 2, with the five typical flows of Heilongjiang→Liaoning (4.82%), Hebei→Beijing (3.48%), Sichuan→Chongqing (2.68%), Jiangsu→Shanghai (2.62%), Heilongjiang→Jilin (2.44%). The main origins include Heilongjiang, Sichuan, Hebei, Henan, Anhui, Shandong, Inner-Mongolia, and the main destinations include Beijing, Shanghai, Xinjiang, Liaoning, Tianjin, Chongqing, and Jilin. 

**Figure 3:**
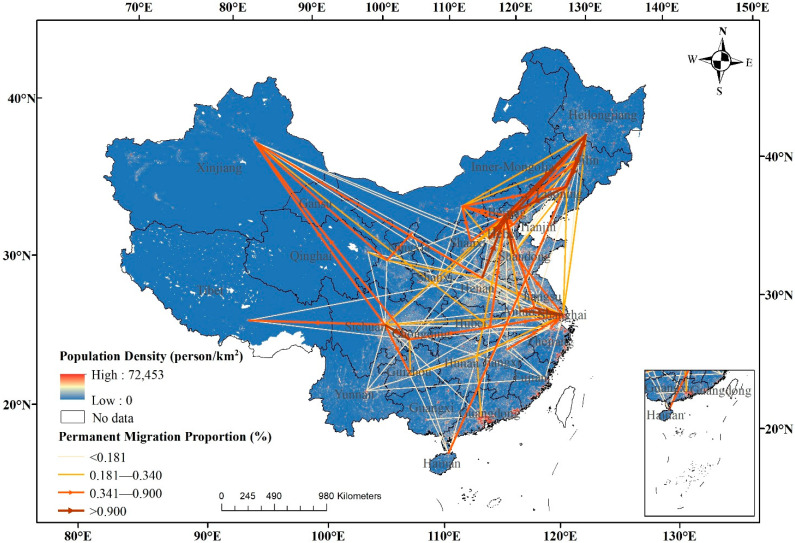
Permanent migration flows of older migrants on the China provincial scale in 2017. Note: The population density data were obtained from Resource and Environment Science and Data Center (https://www.resdc.cn/, accessed on 1 January 2021).

In the flow diagrams of permanent migration proportion, we gathered the number of older migrants willing to settle and obtained the proportion of older migrants with settlement intentions to total samples for each flow. Similarly, a thicker flow represents a higher permanent migration proportion, a thinner flow represents a lower permanent migration proportion, and the arrow represents the permanent migration direction. Obviously, there are fewer thicker flows in the permanent migration pattern of older migrants from Figure 3, in which Heilongjiang→Liaoning flow decreased by 1.24%, Hebei→Beijing flow decreased by 1.48%, Jiangsu→Shanghai flow decreased by 0.83%, Sichuan→Chongqing flow decreased by 1.20%, Heilongjiang→Jilin flow decreased by 1.00%. It should be noted that the origins of Henan, Anhui, and Shandong have undergone drastic changes, with a large number of older people moving out, but few permanent ones. It should be noted that there are numerous older people moving out but few permanently moving out in densely populated origins, such as Henan, Anhui, and Shandong.

Different from adult migrants [55], older migrants presented an uneven agglomeration pattern, including five main types: (1) high permanently emigration: Heilongjiang and Sichuan. Numerous older people move out and settle permanently in destination; (2) medium permanently emigration: Hebei and Inner Mongolia. The main flows from Hebei were moving to Beijing and Tianjin, and the main flows from Inner Mongolia were moving to Shanxi, Liaoning, and Beijing; (3) medium temporarily emigration: Henan, Anhui, and Shandong, which are densely populated provinces in the Central China; (4) high permanently immigration: Beijing, Shanghai, Xinjiang, and Liaoning. Beijing and Shanghai were equipped with the best medical and public services in China. Xinjiang attracted older migrants from the surrounding provinces. Liaoning attracted some older people from Heilongjiang and Jilin; and (5) medium permanently immigration: Tianjin, Chongqing, and Jilin. 

### 3.2. Temperature Effects on Settlement Intentions

To explore temperature effects on settlement intentions, we conducted four models for logistic regression analysis (as shown in Table 2). The cold effect on older migrants was expressed by the origin coldest-month average temperature and destination coldest-month average temperature in Model 1. The heat impact on older migrants was expressed by origin hottest-month average temperature and destination hottest-month average temperature in Model 2. The temperature gap between origin and destination was expressed by coldest-month temperature gap and hottest-month temperature gap in Model 3. The temperature zone effect was expressed by temperature zone spanning in Model 4. The results of models 1 to 4 suggest that climate had an independent role in the settlement intention of older migrants although many control variables play important roles. The upper and lower limits of odds ratio were shown in Table S1 (Supplementary Materials).

(1) Cold effect: Model 1 indicated that low temperature had a great impact on older people’s permanent migration. There was a significant negative correlation (*p* < 0.01) between origin coldest-month average temperature and the settlement intention of older migrants. Older migrants with an origin coldest-month average temperature of −10 °C and below were 2.123 times more likely to move out permanently than those of 0 °C and above. Older migrants with an origin coldest-month average temperature of −10 to 0 °C were 1.303 times more likely to move out permanently than those of 0 °C and above. However, we found no significant correlation between destination coldest-month average temperature and settlement intentions of older migrants.
(2) Heat effect: Similar to Model 1, there was a significant negative correlation (*p* < 0.01) between origin hottest-month average temperature and the settlement intention of older migrants in Model 2, but there was no significant correlation between destination hottest-month average temperature and settlement intention. Older migrants with an origin hottest-month average temperature of 23 °C and below were 2.029 times more likely to move out permanently than those of 28 °C and above. Heat was found not to be statistically related to older immigrants to move out permanently.
(3) Temperature gap effect: In Model 3, we found that the greater the coldest-month temperature gap, the higher the settlement probability, in which the settlement probability with coldest-month temperature gap more than 5 °C is 68.1% higher than that less than 5 °C (*p* < 0.01). There was no significant correlation between hottest-month temperature gap and settlement intentions of older migrants.
(4) Temperature zone effect: Model 4 demonstrated that the probability of older migrants settling in adjacent temperature zones was 29.1% higher than that in the similar temperature zone, while non-adjacent temperature zones were not significant in statistic correlation.

### 3.3. Other Factors Impact on Settlement Intentions

**Figure 4:**
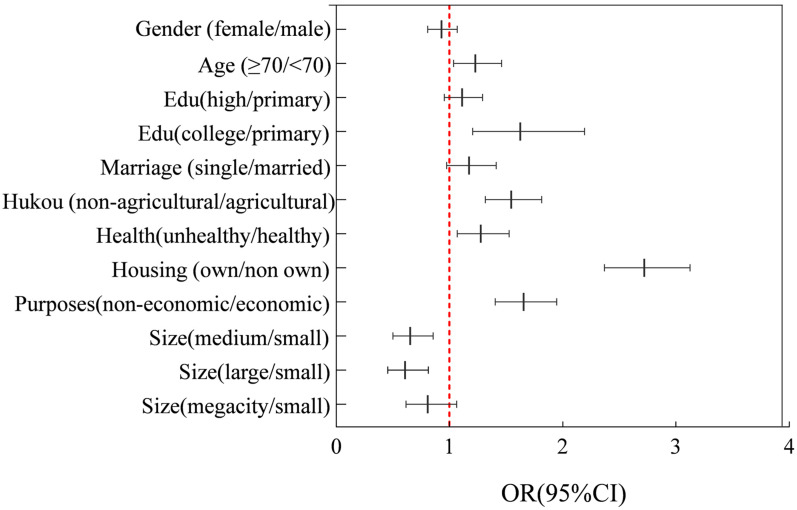
Odds ratio and 95% confidence interval of categorical variables from Model 1.

Figure 4 shows the odds ratio (OR) and 95% confidence interval (CI) of the classified variables affecting older migrants’ settlement intention from Model 1. The dotted line “OR = 1” indicates that there was no difference between the two types of variables, while the value on the right of the dotted line shows a more influential variable than the benchmark variable and the value on the left is the opposite. For classified variables, the greater the OR value, the greater the effect on settlement probability. Specific results are as follows:

(1) Taking below 70 years old as the base group, the 70 and above years old group had the highest willingness to settle in a destination location (OR = 1.230). The willingness to settle improved with education improvement. Older people with “college and above” levels of education were 62.6% more likely than “primary and below” to settle in a destination location. Unmarried, divorced, or widowed older migrants were 17.4% more likely to settle than married ones. Similar to previous studies, the older migrants’ settlement intentions with non-agricultural Hukou were 54.6% higher than that of agricultural Hukou. Considering physical health, unhealthy older people were more likely to settle (OR = 1.278) due to a desire to escape the cold climate and they need care from others.
(2) The higher the ratio of expenditure to income, the higher the willingness to settle in the destination. Older migrants who own housing in the destination were 2.720 times than those non-owning to settle at a destination. Owning housing played a particularly important role in the willingness to settle down, especially for Chinese people because the concept of “home” and “home attachment” in turn tied older migrants to their destinations [56]. In the continual pursuit of “home away home”, real estate as the carrier of “home” promoted the reconstruction of place dependence and place identification.
(3) There was a negative correlation between older migrants’ settlement intention and logarithmical normalization distance consistent with basic assumptions of the gravity model. The settlement probability decreased by 10.5% for each increase of 1% in distance between origin and destination. Non-economic older migrants were 65.4% more likely to settle at a destination than older migrants who were working or in business. The longer an older people migrant lived in a destination, the higher the settlement probability and if they stayed at the destination for one more year, the settlement probability increased by 6.9%.
(4) The older migrants living in small cities had the highest willingness to settle down, while there was no significance in megacities. Small cities were characterized by lower migration and living costs. Healthcare access was a key consideration in settlement decisions and it was also confirmed by the number of beds per thousand people, specifically, the settlement probability increased by 12.4% for an increase of one bed per thousand people. Compared with the high correlation between young migrants and GDP per capita, there was no significant employment effect on older migrants.

## 4. Discussion

### 4.1. Temperature Effect on Older Permanent Migration

Existing studies have suggested that extreme low temperature and extreme high temperature can prompt residents to migrate, with an expression of a nonlinear relationship. However, this study presents more nuanced results. We found that heat did not increase the settlement probability and cold was the main driving force for settlement of older migrants. The settlement probability increases as the origin coldest-month average temperature decreases, and the older migrants from origins with a coldest-month average temperature of −10 °C and below are most likely to move out permanently, showing an obvious characteristic of “escaping extreme low temperature”. The migration pattern of “escaping extreme low temperature” in China is similar to that in the northern United States, Canada, and Europe [38]. For example, Shariff (2021) found that 3% of Ontarians aged 65 or more migrate to Florida for escaping Canadian winters [1]. Casado-diaz (2004) proposed that retirees move from northern European to the southern European sunbelt to avoid the cold, wet northern winters [57]. However, Nawrotzki (2017) revealed that the association between adverse climate conditions and migration is positive only for wealthy districts, and poor districts are characterized by climate-related immobility, such as Zambia in Africa [58].

In addition, some studies suggested that the comfort temperature (temperature difference between July and January) of destination is the driver for the young migrants [30]. Based on the regression analysis of the comfort temperature difference between origin and destination, we found that the results were not significant for older migrants. Compared with “escaping extreme high temperature” or “pursuing comfortable temperature”, older migrants are more likely to “escape extreme low temperatures”. One potential explanation could be the difference of temperature-related impacts between older people and young people [59]. For instance, older people’s thermal sensation was found to be generally 0.5 scale units lower than young people’s [60], and extreme low temperature increases older people’ risks of getting cardiovascular and cerebrovascular diseases.

The temperature of destination may affect the temporary or cyclic migration for older people, typically represented by “Snowbirds” or seasonal migrants. For the settlement intention of older people, this paper demonstrates that relative temperature rather than absolute temperature of destination has a significant effect. If the coldest-month average temperature in the destination place is 5 °C higher than that in the origin place, the probability of permanent migration will be greatly increased. Instead of a generalized intention of “escaping extreme low temperature”, older migrants tend not to settle in an area with a specific temperature range but to settle in a destination place with warmer winter than their origin place. Spatially, geographically adjacent temperature zones are generally an optimal choice for the older migrants to settle, along with a better but small climate difference. Adjacent temperature zones greatly alleviate the low winter temperatures and have similar cultural environments and lower migration costs. In addition, older migrants tend to settle temporarily or seasonally when across two or more temperature zones.

### 4.2. Policy Implications on Urban Development

The permanent migration flow network presents an uneven agglomeration pattern in space. There are three main emigration areas: high permanently emigration, including Heilongjiang and Sichuan; medium permanently emigration, including Hebei and Inner Mongolia; medium temporarily emigration, including Henan, Anhui, and Shandong. There are two main immigration areas: high permanently immigration, including Beijing, Shanghai, Xinjiang, and Liaoning; medium permanently immigration, including Tianjin, Chongqing, and Jilin. The transportation infrastructures, including railways and roads, play a facilitating role on regional migration and tourism [61,62], which has been proved by the dense migration flows in the developed transportation regions in Figure 2. However, an area with developed transportation does not mean a higher willingness to settle (as shown in Figure 3). The immigration and emigration of older migrants should be considered by the main origins and destinations, especially in the allocation of public service resources and urban planning [63]. We have deepened the understanding of the migration and permanent migration of older migrants. 

Considering the temperature impact on regional migration and settlement intention of older people, our findings will inform policy makers of facilitating older migrants who are suffering from negative temperature effects to migrate if needed. More attention needs to paid to those older people who are living in cities with the coldest-month temperature below −10 °C especially in severe cold zone. Their main settlement destinations are small cities with a large coldest-month average temperature gap between origin and destination in adjacent temperature zones, and with more hospital beds. Encouraging to purchase real estate or providing housing subsidies at the destination has the potential to facilitate older migrants relocating to the temperature-friendly environment through migrations. 

## 5. Conclusions

Based on the China Migrants Dynamic Survey in 2017, this paper explored the relationship between temperature and settlement intentions of older migrants using spatial analysis and binary logical regression. The results showed that cold has a significant impact on settlement intentions of older people, while heat has no significant impact. Older people trapped in the area with coldest-month average temperature below −10 °C need to be paid more attention. In addition, individual, family, migration, and destination factors also play a role in promoting older migrants to settle in destination. Interregional migration policies, especially in main emigration and immigration areas, need to be further studied to increase the well-being of the elderly and improve the efficiency of public service utilization. 

However, there are some limitations in this study. First, the odds ratios only approximate probability when the outcome is rare. Second, we only estimated the model parameters using data in 2017. Finally, the precipitation, elevation, and other indicators of climate have not been adopted in this research. Furthermore, we will focus on the outcome and interpretation of the odds ratio, collect panel data of multiple years for in-depth research, and explore the relationship between more climate indicators and older migrations. 

## Figures and Tables

**Table 1:** Definitions and descriptive statistics of variables.

Variable	Description	Dummy: Percentage Frequencies	Continuous: Mean Value
Dependent variable			
SI	Settlement intention of the older migrants (if migrant i decides to settle in city j = 1; otherwise, 0).	1:0~58:42	
Key independent variables			
OCT	Cold: Origin coldest-month average temperature (dummy: <−10 °C = 0; −10 °C–0 °C = 1; >0 °C = 2).	0:1:2~30:27:43	
DCT	Cold: Destination coldest-month average temperature (dummy: <−10 °C = 0; −10 °C–0 °C = 1; >0 °C = 2).	0:1:2~25:35:40	
OHT	Heat: Origin hottest-month average temperature (dummy: <23 °C = 0; 23 °C–28 °C = 1; >28 °C = 2).	0:1:2~17:68:15	
DHT	Heat: Destination hottest-month average temperature (dummy: <23 °C = 0; 23 °C–28 °C = 1; >28 °C = 2).	0:1:2~17:63:20	
GCT	Gap: Coldest-month temperature gap between origin and destination (dummy: <5 °C = 0; ≥5 °C = 1).	0:1~87:13	
GHT	Gap: Hottest-month temperature gap between origin and destination (dummy: <1 °C = 0; ≥1 °C = 1).	0:1~79:21	
Spanning	Temperature zone spanning between O-D (dummy: similar = 0; adjacent = 1; non-adjacent = 2).	0:1:2~70:25:5	
Control variables			
Gender	Gender of the respondent (dummy: Male = 1; female = 0).	1:0~58:42	
Age	Age of the respondent (dummy: <70 = 1; ≥70 = 0).	1:0~78:22	
Edu	Education level (dummy: primary and below = 0; high school and below = 1; college and above = 2).	0:1:2~48:48:6	
Marriage	Marital status (dummy: married = 1; single = 0).	1:0~84:16	
Hukou	The property of Hukou (dummy: non-agricultural = 1, agricultural = 0).	1:0~42:58	
Health	Health status (dummy: healthy = 1; unhealthy = 0).	1:0~81:19	
Exp/Inc	The proportion of family monthly income to the monthly family expenditure of the respondent (continuous; rate).		2.127
Housing	The property of the house (dummy: own = 1; non own = 0).	1:0~55:45	
LnD	Logarithmical distance in O–D. (continuous).		0.125
Purposes	Purposes (dummy: non-economic = 1; economic = 0).	1:0~63:37	
Time	Residence time after migration (continuous; years).		8.950
Size	City size (dummy: small city (<1 million residents) = 0; medium city (1–5 million) = 1; large city (5–10 million) = 2; megacity (>10 million) = 3).	0:1:2:3~7:39:21:33	
GDP	Per capita gross domestic product (continuous; 10^4^ yuan).		7.561
Beds	Hospital beds per thousand people (continuous; beds).		5.672

**Table 2:** The results of odds ratio in the binary logistic model.

Variable	Model 1 Cold Effect	Model 2 Heat Effect	Model 3 Temperature Gap Effect	Model 4 Temperature Zone Effect
Dependent variable				
SI	Settlement intention of the older migrants.			
Key independent variables				
OCT (>0 °C = reference)				
<−10 °C	2.123 ***			
−10–0 °C	1.303 ***			
DCT (<−10 °C = reference)				
<−10 °C	0.811			
−10–0 °C	1.043			
OHT (>28 °C = reference)				
<23 °C		2.029 ***		
23–28 °C		1.758 ***		
DHT (>28 °C = reference)				
<23 °C		1.082		
23–28 °C		1.093		
GCT (<5 °C = reference)			1.681 ***	
GHT (<1 °C = reference)			1.158	
Spanning (similar = reference)				
Adjacent				1.291 ***
Non-adjacent				1.056
Control variables				
Gender (male = reference)	0.930	0.953	0.950	0.955
Age (<70 = reference)	1.230 **	1.218 **	1.257 ***	1.226 **
Edu (primary = reference)				
High school	1.112	1.164 **	1.137 *	1.135 *
College	1.626 ***	1.703 ***	1.612 ***	1.616 ***
Marriage (married = reference)	1.174 *	1.165	1.171 *	1.176 *
Hukou (agricultural = reference)	1.546 ***	1.658 ***	1.594 ***	1.676 ***
Health (healthy = reference)	1.278 ***	1.288 ***	1.331 ***	1.342 ***
Exp/Income	1.819 ***	1.877 ***	2.004 ***	1.970 ***
Housing (non own = reference)	2.720 ***	2.733 ***	2.693 ***	2.697 ***
Purposes (economic = reference)	1.654 ***	1.661 ***	1.657 ***	1.679 ***
Time	1.069 ***	1.070 ***	1.070 ***	1.069 ***
Lnd	0.895 ***	0.893 ***	0.816 ***	0.857 ***
Size (small city = reference)				
Medium city	0.655 ***	0.708 **	0.613 ***	0.657 ***
Large city	0.609 ***	0.728 **	0.558 ***	0.633 ***
Megacity	0.809	0.868	0.755 **	0.811
GDP	1.012	1.022 **	1.006	1.009
Beds	1.124 ***	1.076 **	1.150 ***	1.114 ***

Note: * *p* < 0.1, ** *p* < 0.05, *** *p* < 0.01.

## Data Availability

The China Migrant Dynamic Survey (CMDS) data were obtained from Migrant Population Service Center, National Health Commission, with open access (https://www.chinaldrk.org.cn/, accessed on 1 January 2021).

## Supplementary Materials

The following supporting information can be downloaded at: https://www.mdpi.com/article/10.3390/ijerph19084896/s1, Table S1. Odds ratio and its upper and lower limits in logistic model.
