# Supplementary material for: Exploring the Climate Temperature Effects on Settlement Intentions of Older Migrants: Evidence from China
Source: Int J Environ Res Public Health. 2022 Apr 18;19(8):4896. doi: 10.3390/ijerph19084896 (PMC9028836; doi:10.3390/ijerph19084896)
Supplement: Supplementary file 1 [file ijerph-19-04896-s001.zip › ijerph-1659040-supplementary.pdf]

Supplementary Material

Table S1. Odds ratio and its upper and lower limits in logistic model.

Variable	Model 1: Cold effect			Model 2: Heat effect			Model 3: Temperature gap effect			Model 4:Temperature zone effect		
	OR	-	+	OR	-	+	OR	-	+	OR	-	+
**Dependent variable**												
SI	Settlement intention of the older migrants.											
**Key independent variables**												
OCT (>0℃=reference)												
<-10 ℃	**2.123*****	1.705	2.643									
-10-0 ℃	1.303***	1.072	1.585									
DCT (<-10℃=reference)												
<-10 ℃	0.811	0.635	1.036									
-10-0 ℃	1.043	0.864	1.259									
OHT (>28℃=reference)												
<23℃				**2.029*****	1.561	2.636						
23-28℃				1.758***	1.437	2.152						
DHT (>28℃=reference)												
<23℃				1.082	0.828	1.413						
23-28℃				1.093	0.903	1.323						
GCT (<5℃=reference)							**1.681*****	**1.366**	**2.069**			
GHT (<1℃=reference)							1.158	0.987	1.359			
Spanning (similar =reference)												
Adjacent										**1.291*****	1.100	1.518
Non-adjacent										1.056	0.774	1.443
**Control variables**												
Gender (male=reference)	0.93	0.808	1.069	0.953	0.829	1.095	0.95	0.827	1.092	0.955	0.831	1.097
Age (<70=reference)	1.230**	1.037	1.460	1.218**	1.027	1.446	1.257***	1.060	1.491	1.226**	1.035	1.454

Edu (primary =reference)										***		
High school	1.112	0.956	1.292	1.164**	1.002	1.353	1.137*	0.979	1.321	1.135*	0.978	1.318
College	1.626***	1.205	2.194	1.703***	1.262	2.298	1.612***	1.196	2.173	1.616***	1.200	2.177
Marriage (married=reference)	1.174*	0.975	1.414	1.165	0.968	1.402	1.171*	0.973	1.409	1.176*	0.978	1.414
Hukou (agricultural=reference)	1.546***	1.318	1.815	1.658***	1.414	1.943	1.594***	1.360	1.868	1.676***	1.432	1.962
Health (healthy=reference)	1.278***	1.070	1.527	1.288***	1.079	1.537	1.331***	1.116	1.588	1.342***	1.125	1.601
Exp/Income	1.819***	1.386	2.387	1.877***	1.432	2.461	2.004***	1.530	2.623	1.97***	1.506	2.577
Housing (non own=reference)	2.720***	2.369	3.124	2.733***	2.382	3.137	2.693***	2.348	3.089	2.697***	2.352	3.093
Purposes (economic=reference)	1.654***	1.405	1.947	1.661***	1.412	1.954	1.657***	1.409	1.949	1.679***	1.429	1.974
Time	1.069***	1.059	1.079	1.070***	1.060	1.080	1.070***	1.060	1.080	1.069***	1.059	1.079
Lnd	0.895***	0.834	0.960	0.893***	0.832	0.957	0.816***	0.755	0.881	0.857***	0.787	0.934
Size (small city=reference)												
Medium city	0.655***	0.501	0.856	0.708**	0.543	0.923	0.613***	0.471	0.798	0.657***	0.506	0.853
Large city	0.609***	0.456	0.814	0.728**	0.541	0.980	0.558***	0.420	0.742	0.633***	0.479	0.837
Megacity	0.809	0.616	1.063	0.868	0.654	1.153	0.755**	0.577	0.986	0.811	0.621	1.059
GDP	1.012	0.992	1.032	1.022**	1.000	1.043	1.006	0.987	1.027	1.009	0.989	1.029
Beds	1.124***	1.061	1.190	1.076**	1.016	1.140	1.150***	1.086	1.217	1.114***	1.053	1.178

Notes: “-“ represents the lower limit of 95% CI, “+” represents the upper limit of 95% CI. * p <0.1, ** p < 0.05, *** p < 0.01.
